# Microtubule plus-end tracking Adenopolyposis Coli negatively regulates proplatelet formation

**DOI:** 10.1038/s41598-018-34118-y

**Published:** 2018-10-25

**Authors:** C. Strassel, S. Moog, L. Mallo, A. Eckly, M. Freund, C. Gachet, F. Lanza

**Affiliations:** Université de Strasbourg, INSERM, EFS Grand Est, BPPS UMR-S 949, FMTS, F-67000 Strasbourg, France

## Abstract

Platelets are produced upon profound reorganization of mature megakaryocytes (MK) leading to proplatelet elongation and release into the blood stream, a process termed thrombopoiesis. This highly dynamic process requires microtubules (MT) reorganization by mechanisms that are still incompletely understood. Adenomatous polyposis coli (APC) is a microtubule plus-end tracking protein involved in the regulation of MT in a number of cell systems and its inactivation has been reported to alter hematopoiesis. The aim of our study was to investigate the role of APC in megakaryopoiesis and the final steps of platelet formation. Down-regulation of APC in cultured human MK by RNA interference increased endomitosis and the proportion of cells able to extend proplatelets (68.8% (shAPC1) and 52.5% (shAPC2) vs 28.1% in the control). Similarly an increased ploidy and amplification of the proplatelet network were observed in MK differentiated from Lin- cells of mice with APC-deficiency in the MK lineage. In accordance, these mice exhibited increased platelet counts when compared to wild type mice (1,323 ± 111 vs 919 ± 52 platelets/µL; n = 12 p 0.0033**). Their platelets had a normal size, ultrastructure and number of microtubules coils and their main functions were also preserved. Loss of APC resulted in lower levels of acetylated tubulin and decreased activation of the Wnt signaling pathway. Thus, APC appears as an important regulator of proplatelet formation and overall thrombopoiesis.

## Introduction

Blood platelets which are produced at 10^11^ per day play a critical role in primary hemostasis by preventing blood loss following vascular damage. Understanding the mechanisms that govern their production is of great interest both on a fundamental standpoint and for future transfusion applications. Platelet biogenesis is ensured through a highly orchestrated process whereby hematopoietic stem cells (HSCs) give rise to progenitors that progressively commit to the megakaryocytic lineage producing immature megakaryocytes (MK)^1–3^. MK maturation involves an increase in DNA content (up to 64 N) via endomitosis and an enlargement of the MK cytoplasm associated with the development of an extensive demarcation membrane system and the production of numerous alpha and dense granules^1^. Fully mature MK intimately associated with bone marrow sinusoidal endothelium extend cytoplasmic projections, the proplatelets, following an extensive cytoskeletal remodeling which will further reorganize in the circulation under shear to liberate individual platelets^4,5^.

Imaging studies of *in vitro* differentiated MK have shown that microtubules (MT) power both proplatelet extension and organelle trafficking into the future platelets^6^. MT are dynamic hollow polymers consisting in the assembly of α- and β-tubulin heterodimers. By controlling their organization and stability, MT can adapt to a diverse array of cellular functions. The MT dynamic behavior is controlled through a variety of MT- associated proteins (MAPs). Interaction of tubulins to specialized MAPs is suspected to govern MT assembly into a unique and platelet characteristic circular marginal band. Some MAPs, are specifically localized at the ends of growing MT and are called plus-end tracking proteins (+TIPS). This family of proteins includes Clip170, EB1, Clasp, MCAK, dynein/Dynactin, kinesin and *Adenomatous polyposis coli* (APC)^7^.

APC which is best known for its role as a tumor suppressor, involved in the development of colorectal cancer upon mutagenesis, has been shown to promote MT polymerization and to protect MT from shrinking^8–10^. Interestingly, it has been observed that APC knock-down in dorsal root ganglion (DRG) neurons leads to microtubule looping in the growth cone^11^ an arrangement reminiscent of the marginal band of platelets and proplatelet tips. Referring to the hematopoietic lineage, APC insufficiency leads to ineffective hematopoiesis resulting in exhaustion of the myeloid progenitor pool but its specific role in the MK lineage and platelet biogenesis is unknown^12^. In this study we have explored the role of APC in megakaryopoiesis and the final steps of platelet formation by performing RNA interference in cultured MK and by studying mice with a MK-restricted APC deficiency.

## Material and Methods

### HR35 and APC^−/−^ mice

HR35 mice expressing GFP in all tissues have been described previously^13^. These mice were used in shRNA knockdown studies to obtain a negative control (shGFP) lacking off target effect. *Apc*^fl/fl^ mice carrying a conditionally targeted allele of *Apc* following insertion of a pair of loxP sites into introns 13 and 14 were obtained from B.O Williams (Van Andel Research Institute, Grand Rapids, MI). *Apc*^fl/+^ mice were crossed with transgenic mice selectively expressing cre-recombinase in the megakaryocyte lineage, under control of the *pf4* promoter^14^. Mice were intercrossed to obtain littermate mice homozygous for the wild-type (*Apc*^+/+^) and recombined allele (*Apc*^−/−^).

### Lentiviral short hairpin RNA knockdown

For knockdown in Lin- cells we used shRNA lentiviral transduction particles. The set of shAPC and shGFP sequences were designed by by GeneCopoeia (Rockville, MD) and inserted into pLKO1 vector containing the U6 promoter. Lentivirus particles were produced as described previously by Strassel *et al*.^15^.

### Culture of mouse bone marrow Lin^−^ progenitor cells

Mouse bone marrow Lin^−^ cells were isolated and cultured as described previously^16^. In transduction studies, freshly isolated Lin^−^ cells were incubated for 4 hours with lentiviral particles added at a MOI of 10, cells were then washed and cultured under standard conditions. The percentage of MK extending proplatelets was determined in the culture wells by phase-contrast microscopy. In each culture, at least 600 MK were analyzed and images were acquired using a Zeiss Axio Vert.A1 microscope with a 20x objective (Marly le roi, France).

### RT-PCR

To evaluate the expression of *Apc*, total RNA was isolated from cultured Lin^−^ cells from D0 to D4 and subjected to RT-PCR with *Apc*-specific primers (F:ACAAGACGGCAGCTGGAGTATGAA, R:TGGATCCTGGCTATTCTTCGCTGT), amplified bands were separated by agarose gel electrophoresis and their intensity was measured using GelDoc EZ System. Expression levels were normalized to that of 18S.

### Flow cytometry analysis

#### Determination of ploidy levels

The ploidy level was determined using propidium iodide^17^ and analyzed using a Gallios flow cytometer (Beckman Coulter, Villepinte, France).

#### Determination of platelet number

Culture-derived platelets were released following successive pipetting following addition of 1 μM PGE1 and 0.02 U/mL apyrase in the culture medium and platelets were separated on a BSA gradient as previously described^18^. CD41/CD42b double positive events, having the same scattering properties as circulating blood platelets, were counted as platelet-like particles.

### Immunofluorescence microscopy

Cultured MK were fixed in 4% paraformaldehyde (PFA) for 15 min and cytospun onto poly l-lysine-coated slides. The cells were then permeabilized for 15 min with 0.05% saponin in PBS containing 0.2% BSA and incubated sequentially for 30 min with 10 μg/mL of RAM.1-488 in the same buffer and the nuclei were counterstained with DAPI (Molecular Probes, Thermo Fisher Scientific, UK). The cells were washed thoroughly at each step. The slides were mounted in Mowiol (Mountant, Permafluor, Thermo Fisher Scientific, UK) for examination and proplatelet counting.

### Differential interference contrast microscopy

Cultured MK were fixed at day 4 for 15 min in 4% PFA and cytospun onto poly l-lysine-coated slides. Differential interference contrast (DIC) images were acquired using a Leica DM4000B microscope (Leica Microsystems, Nanterre, France) with a 20×/0.6 NA lens coupled to a CoolSNAP photometrics HQ camera (Roper Scientific, Ottobrunn, Germany). The surface covered by MK bearing proplatelets was determined in 8 random fields by image analysis with Metamorph software (Molecular Devices, Downingtown, PA).

### Transmission electron microscopy

Cells embedded in Epon were classically processed. Thin sections were cut, stained with uranyl acetate and lead citrate and examined under a CM120 transmission electron microscope (TEM) (FEI, The Netherlands)^17^. MK at stages I, II and III were counted manually on whole transversal sections as previously described^17^. To facilitate quantification, each square of the grids is defined as an area for examination (which equals 16,000 µm^2^).

### Western blotting

Cultured MK were collected at day 3 and subjected to enrichment on a BSA gradient (1.5–3%). Cells (4 × 10^6^ cells) were washed in PBS, centrifuged at 100 *g* for 5 minutes and the pellets were resuspended in Laemmli buffer. Proteins from each sample were separated on 4–15% SDS gels (Bio-Rad, Hercules, CA), blotted onto PVDF membranes and incubated with specific primary antibodies. Specifically, anti-acetylated Tubulin Clone 6-11B-1 (Sigma Aldrich, Darmstadt, Germany), anti-α-Tubulin antibody clone DM1a (Sigma Aldrich, Darmstadt, Germany) and anti β-catenin clone 9581 (Cell signaling Technology, Danvers, MA) were used at 0.1 μg/mL, 1 µg/mL, and at a dilution of 1/1000, respectively. Membranes were then washed and incubated for 1.5 hour at room temperature with 50 ng/mL peroxidase-conjugated goat anti-rabbit or with peroxidase-conjugated goat anti-mouse IgG (Jackson Immuno Research, PA, USA) and 50 ng/mL peroxidase conjugated goat anti GAPDH (Abcam, Paris, France) at room temperature and resolved by Clarity Western ECL Substrate (Bio-Rad, Hercules, CA). Membranes were visualized on a Chemidoc Imaging System (Bio-Rad, Hercules, CA).

### Processing for quantitative real-time RT-PCR

RNA preparations were extracted from mature MK (day 3) differentiated from bone marrow Lin-APC^fl/fl^ progenitors or bone marrow Lin- WT progenitors transduced with shAPC using Rneasy mini kit (Qiagen, Courtaboeuf, France). The concentration of the RNA obtained was determined by using the 2100 BioAnalyzer (Agilent Technologies, Tokyo, Japan).

Human Wnt related target genes expression was quantified by qRT-PCR analysis using the RT^2^ Profiler Wnt signaling targets PCR array according to the manufacturer’s recommendations (Qiagen, Courtaboeuf, France). The GAPDH gene was used as internal control and the 2^−ΔΔCt^ value was used to calculate the gene expression transcript levels.

### Statistical analyses

Results were expressed as the mean (±SEM) and statistical comparisons were performed using an unpaired, two-tailed Student’s *t* test (Prism, GraphPad Software Inc., San Diego, CA) or a One-way ANOVA followed by a bonferroni post-test. P values of less than 0.05 were considered to be statistically significant.

## Results

### APC knockdown increases MK ploidy and proplatelet formation

**Figure 1:**
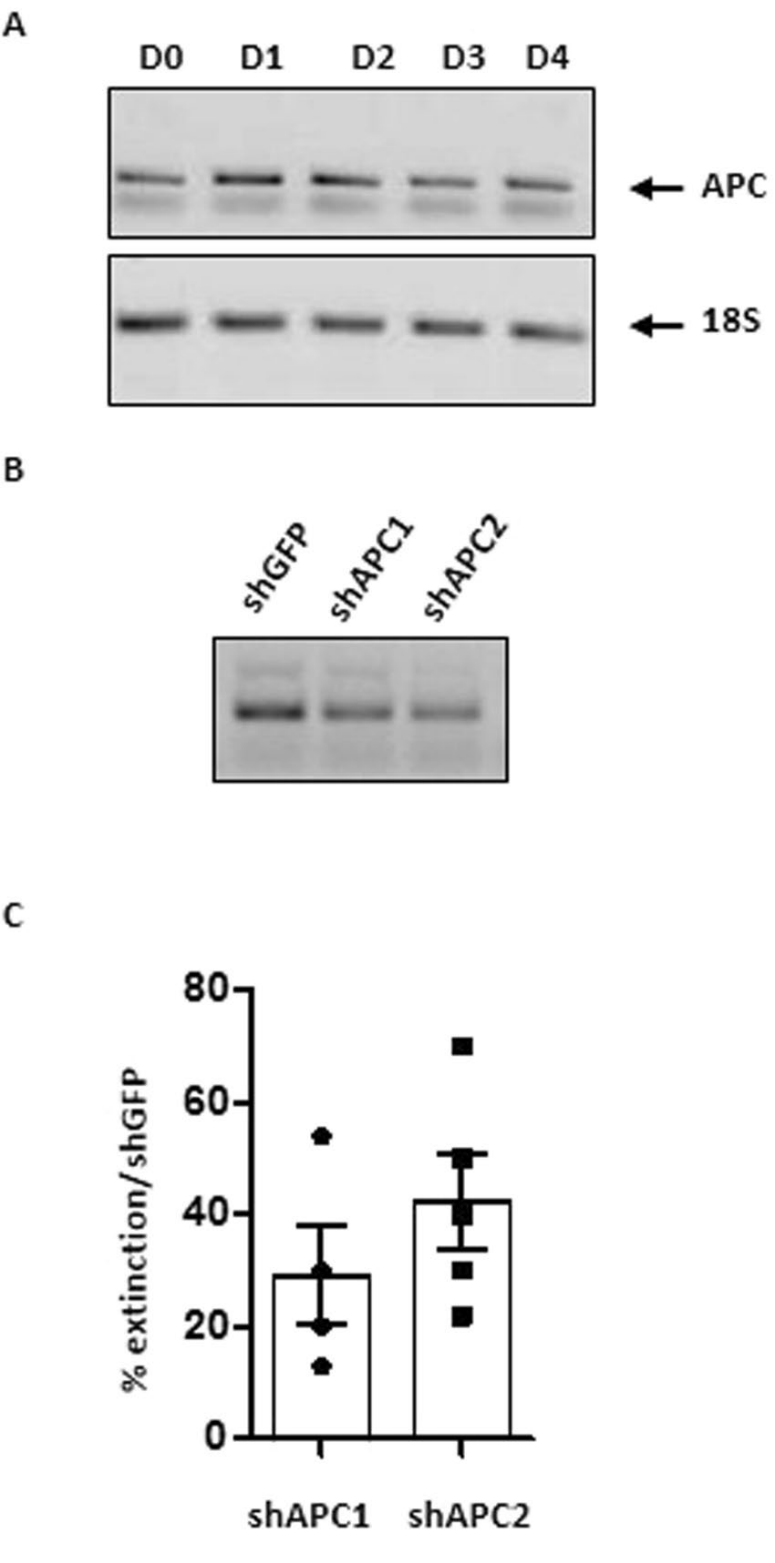
APC expression in mouse cultured MK and its inhibition by RNA interference. **(A)** Lin- progenitors from C57Bl6 mice were cultured for 4 days in MK differentiation conditions as described in the Methods section. Total RNA obtained at the indicated days of culture was subjected to RT-PCR with APC-specific primers and amplified bands were separated by agarose gel electrophoresis. mRNA levels for 20 S are shown as loading controls. **(B)** Lin- progenitors from HR35 mice expressing GFP in all tissues were transduced at day 0 with vectors expressing control GFP shRNA (shGFP) or APC-specific shRNAs (shAPC1, shAPC2) and differentiated into MK. Total RNA was obtained at day 3, subjected to RT-PCR with APC-specific primers and analyzed as in panel A. **(C)** Bar graph representing the decrease in APC mRNA levels at day 3 following shAPC1 and shAPC2 transduction relative to the shGFP control (mean ± SEM from 4 to 5 experiments).

To address the involvement of APC in megakaryopoiesis, we first evaluated its expression in MK differentiated from mouse Lin- progenitors (Fig. 1A). APC transcripts were detected throughout the differentiation and maturation processes from D0 to D4 of culture. We then evaluated the effect of APC knockdown in *in vitro* differentiated MK. Lin- cells expressing GFP were transduced with lentiviral vectors containing shAPC1, shAPC2, or shGFP shRNA as a negative control. shGFP abolished GFP expression in 76% of the cells, establishing efficiency of the procedure (Fig. S1). Under these transduction conditions, shAPC1 and shAPC2 decreased the expression of APC by 29.2 ± 8.9 and 42.4 ± 8.3%, respectively, compared to shGFP control (Fig. 1B,C). shAPC2, the most active shRNA, was then chosen to evaluate the impact on megakaryopoiesis.

**Figure 2:**
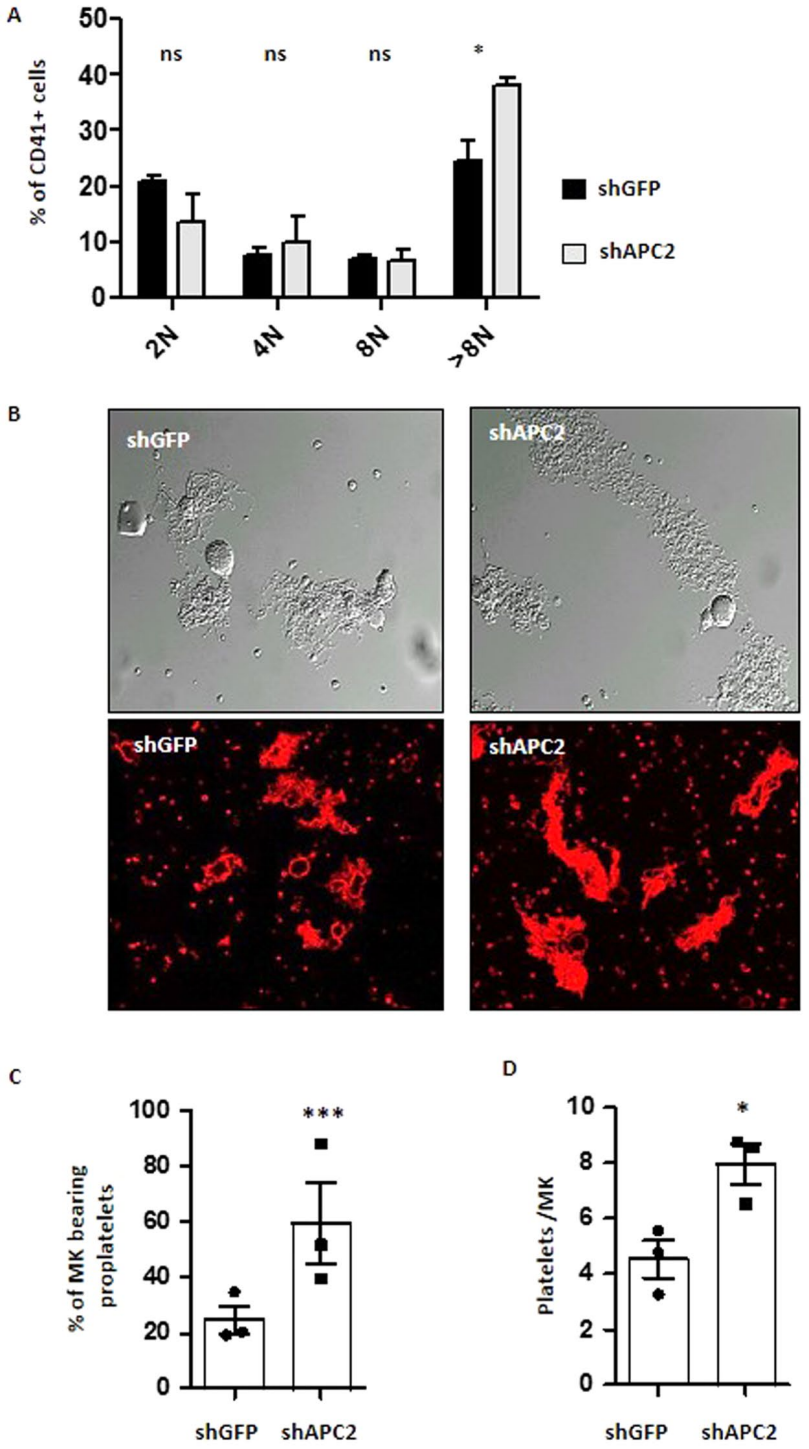
APC knockdown increases MK ploidy, proplatelet formation and platelet production. **(A)** Lin- progenitors from HR35 mice were cultured for 3 days in MK differentiation conditions following transduction with shGFP or shAPC2 and analyzed for their ploidy levels by flow cytometry (mean ± SEM from 3–5 separate experiments). (**B)** Representative images of MK at day 4 of culture examined by DIC microscopy (upper panels) or stained for tubulin (lower panels) showing an increased proportion of proplatelets in the shAPC2 condition. **(C)** Bar graph representing the mean percentage of MK extending proplatelets at day 4 of culture (28.1 ± 3.4% in the control (n = 647 cells from 3 separate cultures) vs 68.9 ± 3.7% for shAPC2 (n = 735 cells from 3 separate cultures; ***p < 0.001). **(D)** Bar graph representing the mean number of platelet elements released from day 4 cultured MK (7.9 ± 0.7 per MK in shAPC2 versus 4.5 ± 0.6 in shGFP control; n = 3; *p = 0.025).

APC knockdown did not modify the number of MK (CD41^+^CD42^+^cells) generated at day 3 (Fig. S2) but influenced their degree of maturation. Firstly, a larger fraction of these MK were of high ploidy in the shAPC2 condition versus control shGFP, with 38.2 ± 3.4 and 24.3 ± 1.4% cells being >8 N, respectively (Fig. 2A). Secondly, there was a 2.5 fold increase in the proportion of proplatelet-producing MK, from 28.1 ± 3.4% in the control (n = 647 cells) to 68.9 ± 3.7% for shAPC2 (n = 735 cells) (Fig. 2C). Thirdly, these proplatelets had a more complex dendritic organization. This resulted in an increased production of platelet-like elements which amounted to 7.9 ± 0.7 per MK in shAPC2 versus 4.5 ± 0.6 (n = 3; *p = 0.0255) in the control (Fig. 2D).

### Mice with APC-deficiency in the MK lineage exhibit increased platelet counts

**Figure 3:**
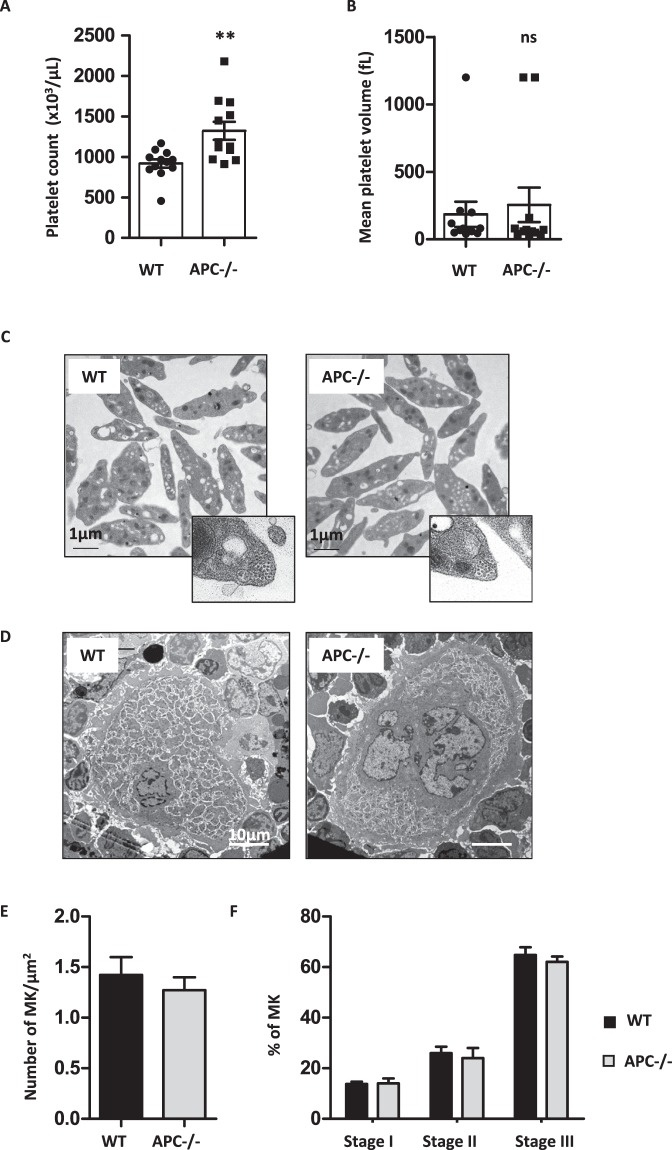
Mice with MK-restricted APC deficiency have increased platelet counts. Mice with APC-deficiency (APC^−/−^) in the MK lineage were generated by crossing *Apc*^*flox/flox*^ mice with *Pf4*-Cre transgenic mice. **(A)** Number of circulating platelets is represented for each individual mouse (1,323 ± 111.10^3^ plts/μL in APC^−/−^ vs 919 ± 52.10^3^ plts/μL in WT mice; n = 12; **p = 0.0033). **(B)** Platelet volumes are represented for each individual mouse (185.8 ± 93.7 fL vs 255.3 ± 127.7 fL; n = 12; ns p = 0.66). **(C)** Representative transmission electron microscopy images showing similar ultrastructures of platelets from wild type and APC^−/−^ mice. Close up views show similar number of MT coils in the two strains. **(D)** Representative TEM images of stage III MK in the bone marrow of wild type and APC^−/−^ mice**. (E)** Morphologically recognizable MK were counted and the numbers expressed as the mean ± SEM per µm^2^. **(F)** The distribution in the different maturation stages was established according to MK morphology^29^.

The above experiments suggested that APC deficiency could translate *in vivo* into an increased number of circulating platelets. Since a complete KO is known to be lethal, we generated a mouse strain with MK-restricted deficiency of APC by crossing *Apc*^*flox/flox*^ mice with *Pf4*-Cre mice^12,14^. *Apc*^*flox/flox*^;*Pf4*-cre mice were obtained at the expected Mendelian frequency, but exhibited premature death due to colon tumor development as previously described^19^. We therefore exclusively used mice aged between 8–14 weeks. Analysis of hematologic parameters revealed a 50% increase in platelet count in *Apc*^*flox/flox*^;*Pf4*-cre (1,323 ± 111.10^3^ plts/μL; n = 12) compared to WT mice (919 ± 52.10^3^ plts/μL; n = 12; **p = 0.0033) (Fig. 3A), with no impact on platelet volume (Fig. 3B), and on erythrocytes and white blood cells counts (Fig. S3). APC-deficient platelets presented a normal ultrastructure with in particular a typical discoid shape and normal number and size of intracellular granules (Fig. 3C). Knowing the role of APC as a + TIP protein, the MT marginal band was observed in more detail but did not reveal any defect in the number and distribution of coils in APC-deficient platelets (Fig. 3C).

### Loss of MK APC increases ploidisation without affecting the ultrastructure

**Figure 4:**
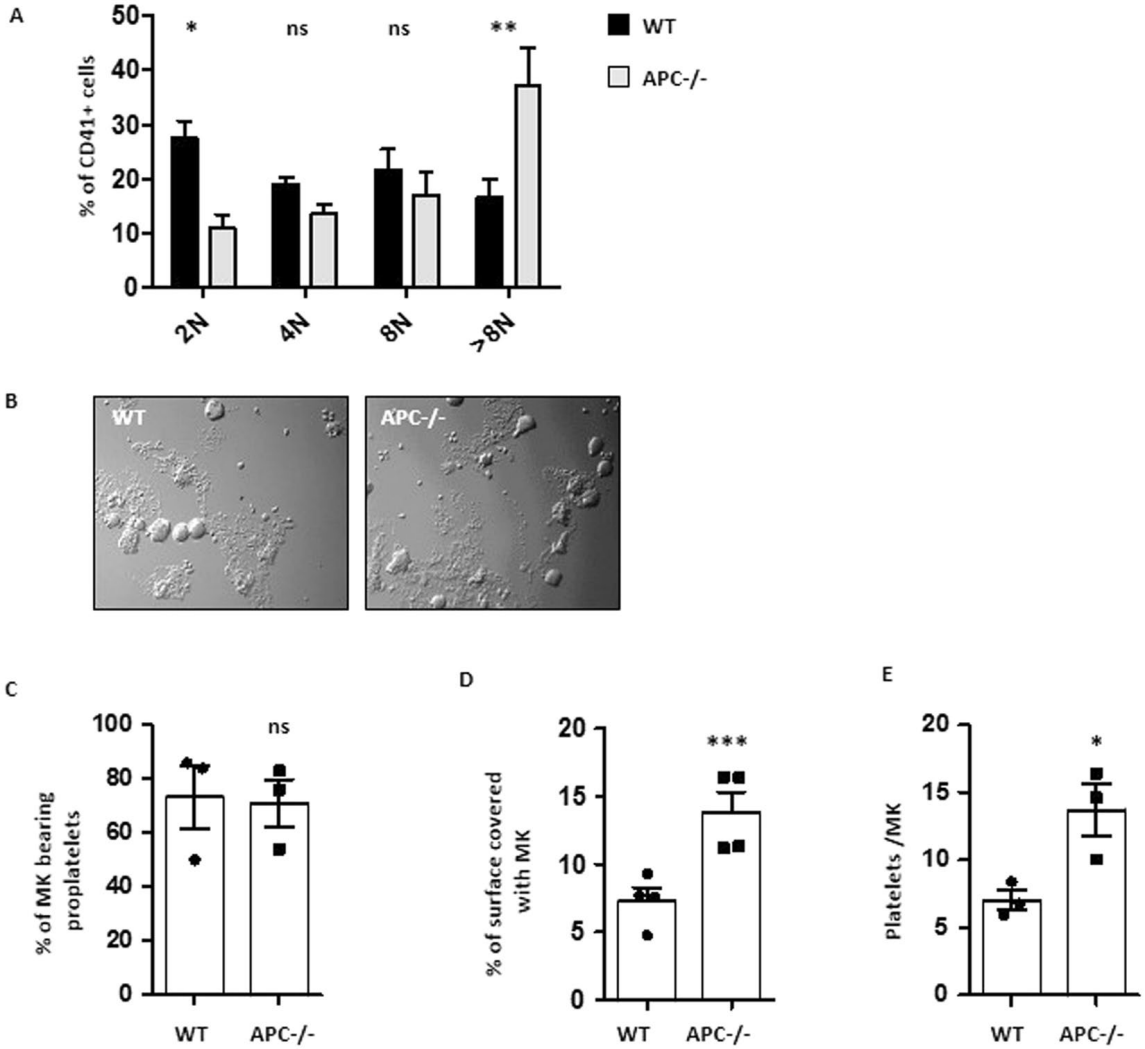
MK cultured from APC^−/−^ mice exhibit increased ploidy, proplatelet branching and platelet production. **(A)** Ploidy analysis of MK (day 3) cultured from Lin- progenitors of wild type or *Apc*^*flox/flox*^;*Pf4*-cre mice (APC^−/−^). **(B)** Representative DIC microscopy images of MK at day 4 of culture. **(C)** Bar graph representing the mean percentage of MK extending proplatelets at day 4 of culture (71 ± 8.7% in APC^−/−^ mice (754 cells) vs 73.3 ± 11.7% in WT mice (826 cells); n = 3; ns) **(D)** Quantification of the surface covered by MK extending proplatelets as a percentage of the area analysed (14.4 ± 1.5% in APC^−/−^ mice vs 7.1 ± 0.7% in WT mice; n = 20–22; ***p < 0.0001). Each point corresponds to a separate culture. **(E)** Bar graph representing the mean number of platelet elements released from day 4 cultured MK (13.7 ± 1.9 in APC^−/−^ vs 7.0 ± 0.7 plts/MK in WT; n = 3; *p = 0.0301).

The increased number of circulating platelets in *Apc*^*flox/flox*^;*Pf4*-cre mice could result from enhanced MK production or maturation. Analysis of the bone marrow showed an equivalent density of morphologically identified MK (1.4 ± 0.2 vs 1.3 ± 0.1 MK/µm^2^, n = 45 and 66) (Fig. 3E). In addition, the distribution between less and more mature MK (stages I-III) was not significantly different in wild type and *Apc*^*flox/flox*^;*Pf4*-cre bone marrow (Fig. 3F). However, an increased ploidy level of bone marrow MK > 8 N (9.9 ± 2.8% vs 16.6 ± 5.6% n = 3–5) was observed in APC-deficient mice (Fig. 4A) replicating the shRNA knockdown effects.

### APC deficiency increases proplatelet network formation

When MK were differentiated from Lin- progenitors, a similar proportion reached the proplatelet stage in *Apc*^*flox/flox*^;*Pf4*-cre as compared to wild type mice (71 ± 8.7% n = 3 (754 cells) vs 73.3 ± 11.7% n = 3 (826 cells)) (Fig. 4B). However, APC-deficient proplatelets exhibited an increased branching and number of tips (Fig. 4C). This was illustrated by an enhanced surface coverage of the well (14.4 ± 1.5% vs 7.1 ± 0.7% n = 22; ***p < 0.0001, 8 random fields (156 µm^2^)) (Fig. 4D) and resulted in an increased number of platelets released in the culture medium (13.7 ± 1.9 vs 7.0 ± 0.7 plts/MK; n = 3; *p < 0.0301) (Fig. 4E).

### Consequence of APC deficiency on MT acetylation and the Wnt/ β-catenin pathway

**Figure 5:**
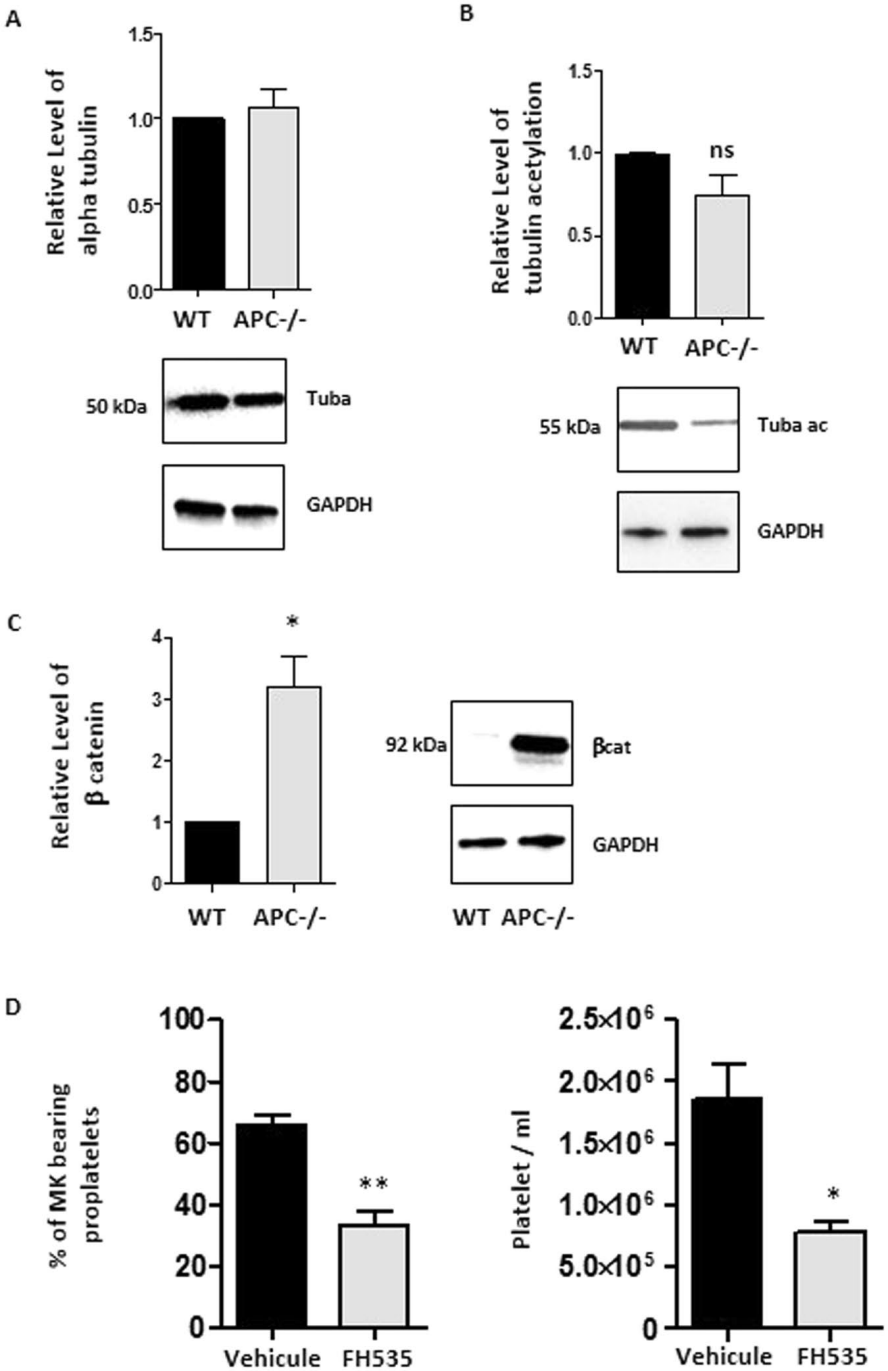
Decreased microtubule acetylation and activation of the pathway in APC-deficient MK. **(A)** Western blot analyses of tubulin acetylation in lysates from cultured MK (day 3) derived from WT mice and APC^−/−^ mice using anti-acetylated tubulin clone 6–11B-1. A decreased level of acetylated tubulin is observed in cultured MK obtained from APC^−/−^ mice and quantified compared to wild type control (n = 3, ns p = 0.11). **(B)** Western blot analyses of total α-tubulin using anti-α-tubulin antibody clone DM1a did not reveal differences between WT mice and APC^−/−^ mice. **(C)** Western blot analyses using an anti–β-catenin antibody showed a dramatic increase in cellular β-catenin levels in APC^−/−^ mice (n = 3, *p = 0.04). (D) Effect of two inhibitors of wnt/ β-catenin signaling on proplatelet formation (65.99 ± 3.02 WT; 33.38 ± 4.25 FH53; n = 3, **p = 0.0036) and platelet production (1.86.10^6^ ± 0.22.10^6^ WT; 0.78.10^6^ ± 0.83.10^6^ FH535, n = 3, *p = 0.02). Bar graph representing the mean ± SEM percentage of MK extending proplatelets and the mean number of platelet elements released from day 4 cultured MK.

Previous work has shown that cells lacking APC harbor MT that are less acetylated in certain areas possibly contributing to their abnormal shape^20^. We therefore explored whether this could also apply to APC-deficient MK. Western blot analysis at day 3 of MK differentiation showed a tendency of lower acetylated tubulin content in *Apc*^*flox/flox*^;*Pf4*-cre mice with normal levels of total α tubulin (Fig. 5A,B).

A well reported consequence of APC deficiency is to prevent β-catenin degradation by the APC-Axin-GSK-3β complex. This was confirmed here in APC-deficient platelets in western blotting assay which exhibited high levels of β-catenin compared to control platelets (Fig. 5C). This accumulation led us to analyze the modulation of the Wnt/β-catenin signaling pathway^21^. Decreased transcripts levels were observed in APC deficient mice for *axin 2* and *ctpb1*, two negative regulators of the canonical pathway, and on the contrary an increased level of *wnt11*, a component of the non-canonical pathway (Fig. S4A). Similar tendencies were noted in MK from WT mice that had been transduced with shAPC2 (Fig. S4B). These results indicate that lack of APC expression results in better activation of the Wnt signaling pathway which could in turn support increased proplatelet formation. In support of this hypothesis we observed that inhibiting Wnt/β-catenin signaling, using an chemical compound FH535^22^, decreased the capacity of cultured MK to extend proplatelets (65.99 ± 3.02 WT; 33.38 ± 4.25 FH53) resulting in the decreased production of platelets in the culture medium by 60% on average (1.86.10^6^ ± 0.22.10^6^ WT; 0.78.10^6^ ± 0.83.10^6^ FH535) (Fig. 5D).

## Discussion

The main finding of this study is that deficiency of APC in MK augments their capacity to extend proplatelets translating into an increased production of platelets. These effects were observed in cultured MK using an RNAi knockdown approach and *in vivo* in mice lacking APC in the megakaryocytic lineage. These results indicate a new role of this multifunctional protein as a negative regulator of the last stages of platelet biogenesis.

Considering the possible mechanisms for the increased platelet production upon APC deficiency two major downstream components of APC signaling can be considered: the β-catenin pathway and microtubules. The implication of microtubules is strongly suggested by previous findings in neuronal cells. The increased capacity to extend proplatelets is reminiscent of the increased axonal branching of cortical neurons in APC conditional knockouts^23^. This later effect appeared to be independent of β-catenin and pointed to a MT-dependent role in neurons. Another parallel between platelet formation and neuron development is the observation that APC knockdown in dorsal root ganglion neurons leads to MT looping in the growth cone^11^, an unusual MT arrangement resembling that occurring during marginal band formation in proplatelet tips^4^. An additional analogy pertains to MT acetylation which was decreased in APC-deficient MK, similar to what has been observed in APC deficient fibroblasts^20^ and is considered to reflect more dynamic MT. The role of tubulin acetylation on platelet biogenesis remains however unclear. Inhibitors of the main deacetylase HDAC6, which increase the level of MT acetylation, have shown an inhibitory effect on megakaryopoiesis *in vitro* and *in vivo*. However, a recent study by Messaoudi K *et al*.^24^ indicates that this is mediated in human cells via acetylation of cortactin and not tubulin. A direct way to address the role of a decreased acetylation, as observed here in APC KO, would be to knock out ATAT1, the main acetylase in platelets, a strategy that is currently under evaluation.

Considering possible mechanisms involving MT, APC can bind to MT plus ends and also interacts with EB1 and the formin mDia (DIAPH) to form a complex which stabilizes the MT growing ends^25^. APC marks the leading edge in adherent cells and favors the linear growth of preexisting MT^25^. This suggests that perturbation of the APC-EB1-mDia1 complex, by removing APC at late stages of MK maturation, could favor a dendritic growth of MT and expand the proplatelet network. Interestingly, RNAi knockdown of mDia1 in MK has been found to increase proplatelet formation similarly to APC knockdown^26^ in line with the proposed MT-dependent mechanism. Additional partners of APC could also play a role such as CLIP-170 together with IQGAP1, which serve to link MT and actin at the leading edge of migrating cells^9,10^. Finally, APC interacts with members of the kinesin family, adding to the array of possibilities for APC to modulate MT functions during proplatelet formation.

The wnt/β-catenin pathway could also facilitate proplatelet formation in view of the marked increase in β-catenin in APC-deficient MK and platelets. In support of this, it has been reported that MK cultured in the presence of wnt3a, which stabilizes β-catenin, more effectively extend proplatelets^21^, but the mechanism remains unclear. We indirectly addressed the hypothesis of a Wnt-β catenin involvement in promoting platelet formation using two well characterized inhibitors of this signaling pathway. We observed that they both inhibit the capacity of cultured MK to extend proplatelets and to produce platelets, results which would be in line with the hypothesis that an increased activity of this pathway would on the contrary promote MK maturation and platelet production. Wnt signaling is not limited to activate transcription of target genes and has also been proposed to regulate actin and MT stability and organization in other cells. For example, wnt3a increases the growth cone size in DRG neurons by a mechanism independent of transcription and acting through a β-catenin pathway directly signaling to the cytoskeleton^11^.

A role of APC in hematopoiesis has been previously documented^12,27^ but our work provides the first indication of a specific role in the MK lineage. APC ablation in the hematopoietic lineage in *Apc*^*flox/flox*^; *Mx1*-cre mice after pIpC injection, resulted in an exhaustion of the HSC pool due to increased cell cycle entry and apoptosis, and a block of multilineage differentiation. The only previous indication of a role of APC in MK has been a report of increased megakaryopoiesis in the spleen of *Apc*^min/+^ mice^28^, which reproduce a human mutation at codon 850 of APC. APC is a large and multidomain protein, and depending on the location of the truncation or mutation, different functional consequences could occur. Deletion of exon 14 as in the present study produces a truncated protein lacking the C-terminal domains including the binding sites for MT and EB1 and also for β-catenin. The *Apc*^min/+^ mice reproduces an antisense mutation resulting in haploinsufficiency of APC and it is not clear if the observed effects result from low protein expression of by production of abnormal forms of APC. In view of the multifunctional nature of APC, its capacity to regulate megakaryopoiesis could clearly occur through multiple mechanisms. Our study clearly indicates that it acts on platelet biogenesis by controlling the final stages of proplatelet formation. This has been shown in separate RNAi and knock out strategies targeting the megakaryocytic lineage. Clear analogies with results obtained in neuronal cells favor a mechanism related to the capacity of APC to modulate MT activity. The only models described to date interfering with MT (TUBB1 KO, MT depolymerization drugs) present with decreased platelet production. The APC-deficient mouse would therefore represent the first indication that platelet production can be positively modulated by acting on the microtubular cytoskeleton.

In conclusion, this study has presented evidence for the role of a non-motor MT associated protein (MAP) in regulating the final stages of MK maturation and platelet biogenesis. The unique mode of MT assembly during thrombopoiesis, leading to marginal band formation in circulating platelets, suggests that a particular set of additional MAPs might be required. Their identification could help us to better decipher the mechanisms of platelet formation and open ways to improve our capacity to produce platelets *in vitro*.

## Electronic supplementary material

supplemental figures

## References

[1] Machlus KR, Italiano JE (2013). The incredible journey: From megakaryocyte development to platelet formation. The Journal of cell biology.

[2] Bluteau D (2009). Regulation of megakaryocyte maturation and platelet formation. Journal of thrombosis and haemostasis: JTH.

[3] Woolthuis CM, Park CY (2016). Hematopoietic stem/progenitor cell commitment to the megakaryocyte lineage. Blood.

[4] Italiano JE, Lecine P, Shivdasani RA, Hartwig JH (1999). Blood platelets are assembled principally at the ends of proplatelet processes produced by differentiated megakaryocytes. The Journal of cell biology.

[5] Lefrancais E (2017). The lung is a site of platelet biogenesis and a reservoir for haematopoietic progenitors. Nature.

[6] Italiano JE, Patel-Hett S, Hartwig JH (2007). Mechanics of proplatelet elaboration. Journal of thrombosis and haemostasis: JTH.

[7] Akhmanova A, Steinmetz MO (2008). Tracking the ends: a dynamic protein network controls the fate of microtubule tips. Nature reviews. Molecular cell biology.

[8] Kita K, Wittmann T, Nathke IS, Waterman-Storer CM (2006). Adenomatous polyposis coli on microtubule plus ends in cell extensions can promote microtubule net growth with or without EB1. Molecular biology of the cell.

[9] Hanson CA, Miller JR (2005). Non-traditional roles for the Adenomatous Polyposis Coli (APC) tumor suppressor protein. Gene.

[10] Aoki K, Taketo MM (2007). Adenomatous polyposis coli (APC): a multi-functional tumor suppressor gene. Journal of cell science.

[11] Purro SA (2008). Wnt regulates axon behavior through changes in microtubule growth directionality: a new role for adenomatous polyposis coli. The Journal of neuroscience: the official journal of the Society for Neuroscience.

[12] Qian Z, Chen L, Fernald AA, Williams BO, Le Beau MM (2008). A critical role for Apc in hematopoietic stem and progenitor cell survival. The Journal of experimental medicine.

[13] Angenieux C (2016). Time-Dependent Decay of mRNA and Ribosomal RNA during Platelet Aging and Its Correlation with Translation Activity. PloS one.

[14] Pertuy F (2015). Broader expression of the mouse platelet factor 4-cre transgene beyond the megakaryocyte lineage. Journal of thrombosis and haemostasis: JTH.

[15] Strassel C (2016). Lentiviral gene rescue of a Bernard-Soulier mouse model to study platelet glycoprotein Ibbeta function. Journal of thrombosis and haemostasis: JTH.

[16] Strassel C (2012). Hirudin and heparin enable efficient megakaryocyte differentiation of mouse bone marrow progenitors. Exp Cell Res.

[17] Strassel C (2009). Intrinsic impaired proplatelet formation and microtubule coil assembly of megakaryocytes in a mouse model of Bernard-Soulier syndrome. Haematologica.

[18] Strassel C (2016). Aryl hydrocarbon receptor-dependent enrichment of a megakaryocytic precursor with a high potential to produce proplatelets. Blood.

[19] Colnot S (2004). Colorectal cancers in a new mouse model of familial adenomatous polyposis: influence of genetic and environmental modifiers. Laboratory investigation; a journal of technical methods and pathology.

[20] Kroboth K (2007). Lack of adenomatous polyposis coli protein correlates with a decrease in cell migration and overall changes in microtubule stability. Molecular biology of the cell.

[21] Macaulay IC (2013). Canonical Wnt signaling in megakaryocytes regulates proplatelet formation. Blood.

[22] Handeli S, Simon JA (2008). A small-molecule inhibitor of Tcf/beta-catenin signaling down-regulates PPARgamma and PPARdelta activities. Molecular cancer therapeutics.

[23] Chen Y, Tian X, Kim WY, Snider WD (2011). Adenomatous polyposis coli regulates axon arborization and cytoskeleton organization via its N-terminus. PloS one.

[24] Messaoudi K (2017). Critical role of the HDAC6-cortactin axis in human megakaryocyte maturation leading to a proplatelet-formation defect. Nature communications.

[25] Wen Y (2004). EB1 and APC bind to mDia to stabilize microtubules downstream of Rho and promote cell migration. Nature cell biology.

[26] Pan J (2014). The formin DIAPH1 (mDia1) regulates megakaryocyte proplatelet formation by remodeling the actin and microtubule cytoskeletons. Blood.

[27] Li W (2013). Apc regulates the function of hematopoietic stem cells largely through beta-catenin-dependent mechanisms. Blood.

[28] You S (2006). Developmental abnormalities in multiple proliferative tissues of Apc(Min/+) mice. International journal of experimental pathology.

[29] Strassel C, Hechler B, Bull A, Gachet C, Lanza F (2009). Studies of mice lacking the GPIb-V-IX complex question the role of this receptor in atherosclerosis. Journal of thrombosis and haemostasis: JTH.

