# Supplementary material for: Microtubule plus-end tracking Adenopolyposis Coli negatively regulates proplatelet formation
Source: Sci Rep. 2018 Oct 25;8:15808. doi: 10.1038/s41598-018-34118-y (PMC6202313; doi:10.1038/s41598-018-34118-y)
Supplement: Supplementary file 1 — supplemental figures [file 41598_2018_34118_MOESM1_ESM.pdf]

Microtubule plus-end tracking Adenopolyposis Coli negatively regulates proplatelet formation

Strassel C[1], Moog S[1], Mallo L [1], Eckly A[1], Freund M[1], Gachet C[1] and Lanza F[1].

[1]**Université de Strasbourg, INSERM, EFS Grand Est, BPPS UMR-S 949, FMTS, F-67000 Strasbourg, France**

Figure S1

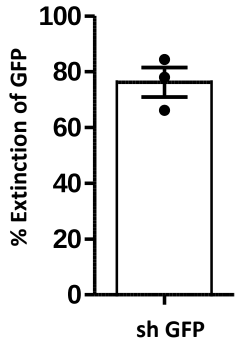

Effect of effect of shGFP on GFP expression. Lin- progenitors from HR35 mice expressing GFP in all tissues were transduced at day 0 with vectors expressing control GFP shRNA (shGFP). Bar graph representing the decrease in GFP transcript in comparison with H35 cells.

Figure S2

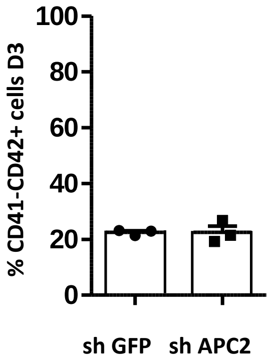

Effect of effect of APC knockdown on the proportion of CD41+ MK. Bar graph representing the mean percentage of the CD41-CD42+ population after 3 days of differentiation following transduction with shGFP or shAPC2 .

Figure S3

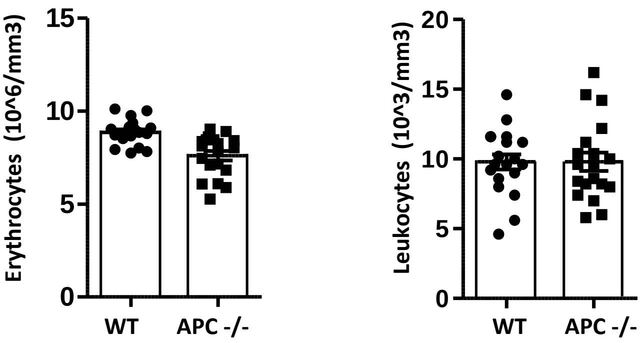

Erythrocytes and leukocytes counts in mice with APC-deficiency (APC-/-) in the MK lineage

Microtubule plus-end tracking Adenopolyposis Coli negatively regulates proplatelet formation

Strassel C[1], Moog S[1], Mallo L [1], Eckly A[1], Freund M[1], Gachet C[1] and Lanza F[1].

[1]**Université de Strasbourg, INSERM, EFS Grand Est, BPPS UMR-S 949, FMTS, F-67000 Strasbourg, France**

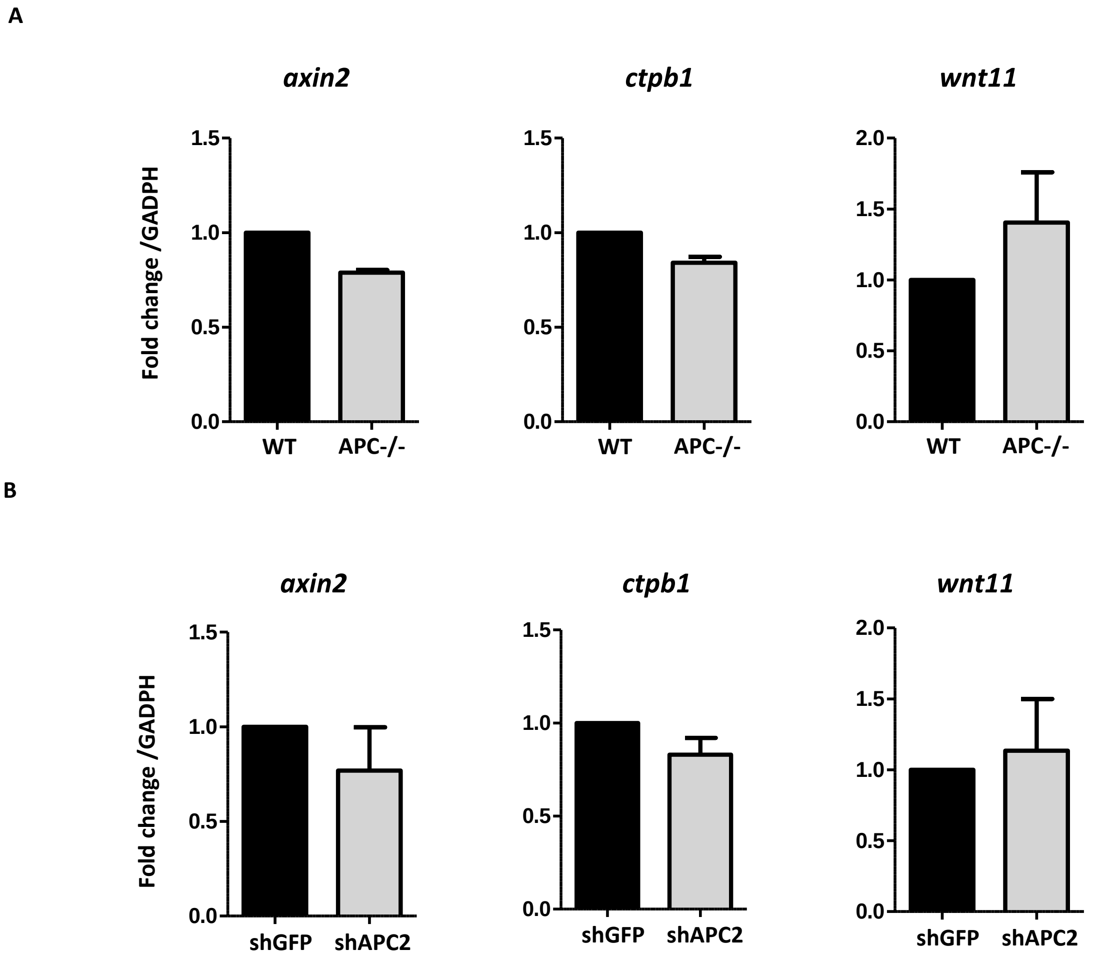

Effect of APC deficiency on wnt target gene expression.
Wnt target gene expression was analyzed by quantitative real-time RT-PCR using the ΔΔct method. RNA were obtained from cultured MK (day 3) derived either from WT and APC-/-mice **(A)** or from MK derived from HR35 mice transduced with shGFP or shAPC2 **(B)**. Results are means of 2 independent experiments. The diagrams show the relative expression of the gene axin1, ctpb1 and wnt11.

Figure S4

Microtubule plus-end tracking Adenopolyposis Coli negatively regulates proplatelet formation

Strassel C[1], Moog S[1], Mallo L [1], Eckly A[1], Freund M[1], Gachet C[1] and Lanza F[1].

[1]**Université de Strasbourg, INSERM, EFS Grand Est, BPPS UMR-S 949, FMTS, F-67000 Strasbourg, France**

Absence (-) ou présence (+) de la cre recombinase

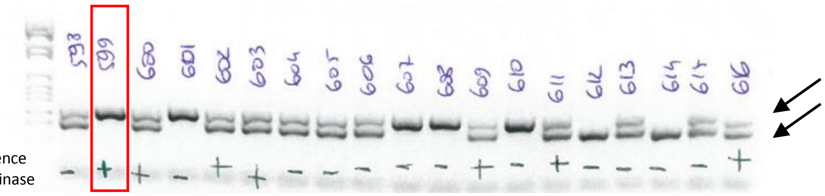

Allèle flox

Allèle WT

Souris KO APC PF4CRE-: Allèle flox et présence de la cre recombinase
